# Randomised Controlled Trial Assessing Head Down Deep Breathing Method Versus Modified Valsalva Manoeuvre for Treatment of Supraventricular Tachycardia in the Emergency Department

**DOI:** 10.5811/westjem.2021.4.51108

**Published:** 2021-07-20

**Authors:** Hoon Chin Lim, Yi-En Clara Seah, Arshad Iqbal, Vern Hsen Tan, Shieh Mei Lai

**Affiliations:** * Changi General Hospital, Accident and Emergency Department, Singapore; † Changi General Hospital, Cardiology Department, Singapore

## Abstract

**Introduction:**

Supraventricular tachycardia (SVT) is commonly encountered in the emergency department (ED). Vagal manoeuvres are internationally recommended therapy in stable patients. The head down deep breathing (HDDB) technique was previously described as an acceptable vagal manoeuvre, but there are no studies comparing its efficacy to other vagal manoeuvres. Our objective in this study was to compare the rates of successful cardioversion with HDDB and the commonly practiced, modified Valsalva manoeuvre (VM).

**Methods:**

We conducted a randomised controlled trial at an acute hospital ED. Patients presenting with SVT were randomly assigned to HDDB or modified VM in a 1:1 ratio. A block randomisation sequence was prepared by an independent biostatistician, and then serially numbered, opaque, sealed envelopes were opened just before the intervention. Patients and caregivers were not blinded. Primary outcome was cardioversion to sinus rhythm. Secondary outcome(s) included adverse effects/complications of each technique.

**Results:**

A total of 41 patients were randomised between 1 August, 2018–1 February, 2020 (20 HDDB and 21 modified VM). Amongst the 41 patients, three spontaneously cardioverted to sinus rhythm before receiving the allocated treatment and were excluded. Cardioversion was achieved in six patients (31.6%) and seven patients (36.8%) with HDDB and modified VM, respectively (odds ratio 1.26, 95% confidence interval, 0.33, 4.84, P = 0.733). Seventeen (89.5%) patients in the HDDB group and 14 (73.7%) from the modified VM group did not encounter any adverse effects. No major adverse cardiovascular events were recorded.

**Conclusion:**

Both the head down deep breathing technique and the modified Valsalva manoeuvre appear safe and effective in cardioverting patients with SVT in the ED.

## INTRODUCTION

Supraventricular tachycardia (SVT) is a common clinical condition seen in the emergency department (ED). It accounts for an an estimated 50,000 visits each year in the USA.^1^ In haemodynamically stable patients presenting with regular narrow complex (QRS ≤ 120 milliseconds) tachycardias, either atrioventricular re-entrant tachycardia (AVRT) or atrioventricular nodal re-entrant tachycardia (AVNRT) is the most common mechanism. In the absence of an established diagnosis at the ED and after ruling out irregular narrow complex tachycardias which are usually due to atrial fibrillation, vagal manoeuvres are recommended as acute therapy for this group of patients.^2^ Previously, we have described the head down deep breathing (HDDB) technique as a reasonable and simple alternative to other vagal manoeuvres for the management of paroxysmal SVT at the ED.^3^ In this study, we assessed the HDDB method with the commonly practised, modified Valsalva manoeuvre (REVERT study)^4^,^5^ and compared the rates of successful cardioversion of SVT to sinus rhythm between the two groups. Our hypothesis is that HDDB is a safe and efficacious method for conversion of stable SVT.

## METHODS

### Study Design

This was a randomised clinical trial assessing HDDB method vs modified Valsalva manoeuvre (VM) for the treatment of SVT presenting to the ED. The study was approved by the Singhealth Centralised Institutional Review Board (CIRB) and received funding from a hospital research grant. All patients provided written informed consent in English. Consent was taken in a standardized manner with provision of study participant patient information sheets. Verbal translation of the consent was provided at the bedside when necessary. Neither patients nor the public were involved in the design, conduct, reporting, or dissemination plans of this study.

### Study Setting and Population

We conducted the study in the ED of an acute hospital in a regional healthcare cluster with an emergency medicine academic clinical programme. The ED has an annual attendance of more than 130,000. Adults 21 years old and above who presented at the ED with paroxysmal SVT on 12-lead electrocardiogram (ECG) during office hours were eligible. They had to be hemodynamically stable, not in imminent danger and able to provide informed consent. The exclusion criteria were as follows: 1) special patient groups: pregnant women, prisoners; 2. hemodynamically unstable patients: low blood pressure: systolic blood pressure (SBP) < 90 milligrams mercury (mm Hg) or mean arterial pressure (MAP) <65 mm Hg, or high blood pressure: SBP ≥ 160 mm Hg and/or diastolic blood pressure (DBP) ≥ 100 mm Hg, ongoing angina pectoris, presence of pulmonary edema; 3) risk from raised intracranial pressure, raised intrathoracic or intra-abdominal pressure; 4) history of hemorrhagic stroke, cerebral arteriovenous malformation, intracranial space-occupying lesion or mass, intracranial aneurysm; 5) history of vascular aneurysm, vascular dissection; 6) unable to perform either manoeuvre (eg, due to inability to lie flat and have legs lifted to assume a head-down tilt position, recent surgery (cardiac surgery or procedures); and 7) use of drugs which inhibit the effects of the vagus nerve, such as atropine. Clinical research coordinators (CRC) and study investigators consented and enrolled patients who were referred to them by emergency physicians.

**Population Health Research Capsule**

What do we already know about this issue?

*Head down deep breathing (HDDB) is a vagal manoeuvre that can be used to cardiovert supraventricular tachycardia (SVT)*.

What was the research question?

*What is the efficacy and safety of HDDB, and how does it compare to the modified Valsalva manoeuvre?*

What was the major finding of the study?

*Our findings suggest that HDDB is safe and effective for cardioversion of SVT. However, further study is needed to confirm this*.

How does this improve population health?

*A simple, non-pharmacological treatment, HDDB, may be self-administered by patients with recurrent SVT. This would be especially useful in low-resource settings*.

### Sample Size Calculation

We estimated the success rate at cardioverting SVT to a sinus rhythm at 43% for modified VM and 20% for the HDDB method. For the study to have 80% power with significance level of 5%, the minimum number of patients to be recruited into each trial therapy was 63 to be able to detect at least a 23% difference of success rate between two arms. To account for a 20% dropout rate, we planned to recruit 75 patients per arm (total 150 patients) into the study.

### Study Protocol

Patients were recruited based on a convenience sampling method due to logistical feasibility. Recruited patients were randomly assigned to either one of the methods, HDDB or modified VM, in a 1:1 ratio. For each assigned treatment method, the patient underwent two attempts with a one-minute interval after each attempt to observe for successful cardioversion. The study ended after two attempts, and this was followed by routine care per clinician discretion.

**Figure 1:**
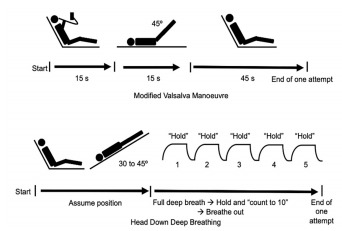
Modified Valsalva manoeuvre and head down deep breathing methods.

The modified VM required the participants to be seated at a 45° angle and perform a standardised strain for 15 seconds. Forced expiration through disposable tubing against a digital manometer at a pressure of 40 mm Hg was maintained for 15 seconds. Following this, the patient was laid flat, and his legs raised to a 45° angle for 15 seconds by the ED staff. Lastly, the participant was returned to a 45° semi-recumbent position for 45 seconds. This comprised one attempt. The HDDB method required the participant to lie on a flat bed with a head-down tilt of 30–45°. Five deep breathing and breath holding repetitions were carried out in one attempt. The patients were instructed to take full deep breaths and hold them by counting to 10 before exhaling. This was to encourage breath holding during full inspiration for as long as the patient could tolerate or by the count of 10 (see Figure 1).

The duration of subject participation was the ED consultation at the time of visit with no subsequent trial scheduled visits or follow-up. Patients could have been recruited more than once if they re-presented at the ED with another episode of paroxysmal SVT fulfilling the inclusion/exclusion criteria of the study. A block randomization sequence was prepared by an independent biostatistician. Serially numbered, opaque, sealed envelopes were prepared according to the randomisation list. Study team members opened the envelopes immediately before the procedure. Patients and treating clinicians were not masked to allocation. The study was stopped when the following occurred: 1) success of manoeuvre with cardioversion to normal sinus rhythm; 2) deterioration of patient’s condition or haemodynamic instability (unstable SVT) which demanded the stoppage of vagal manoeuvre to conduct other treatment methods such as electrical cardioversion; 3) adverse effects of the method and request by the patient to stop the particular intervention; and 4) catastrophic event such as cardiopulmonary arrest, malignant arrhythmia, acute myocardial infarction, or stroke. Adverse events, if any, were reported to the approving CIRB within the stipulated timeframe. All pre- and post-study ECGs were reviewed by VH Tan and HC Lim to confirm that SVT (not atrial fibrillation or atrial flutter) was the initial rhythm and that it was cardioverted to sinus rhythm in successful cases.

### Outcomes

The primary outcome of interest was conversion to sinus rhythm. Secondary outcome(s) studied included adverse effects and /or complications associated with each method.

### Data Analysis

We present collected data as frequency (percentage) for categorical variables. The Shapiro-Wilk test showed normal distribution was met; hence, continuous variables were presented as mean (standard deviation). We compared subject baseline characteristics between groups using chi-square test or Fisher’s exact test for categorical variables, and independent t-test was performed for continuous variables. Analysis was performed in accordance with intention-to-treat principle and missing data were omitted from the analysis. We assessed the association between treatment arm and successful cardioversion as well as adverse event using binary logistic regression model, and results are presented as odds ratios (OR) with 95% confidence intervals (CI). We performed all statistical analyses using SPSS Statistics for Window, version 20 (IBM Corporation, Armonk, NY), and a two-tailed, *P* <0.05 was set to be considered as statistically significant. No interim data analysis was planned.

## RESULTS

During the period 1 August, 2018–1 February, 2020, based on *International Classification of Diseases, 10^th^ Modification* coding, the department attended to 186 patients with SVT. The number of patients who were assessed for eligibility was not recorded. A total of 41 patients were recruited and randomised. No patient was enrolled more than once. The recruitment did not reach the intended sample size of 150 patients due to slow recruitment. This limitation was then compounded by challenges related to policy changes amid the COVID-19 pandemic which resulted in cessation of all CRC activities in the department. Due to the small sample size, inadequate statistical power prevented us from conducting an effective comparison between the two methods, and the study findings are hereby descriptively analyzed.

Among the 41 patients randomised, three (one in the HDDB group and two in the modified VM group) spontaneously cardioverted before receiving the allocated treatment. They were excluded from the final analysis. Two cases in the modified VM group (DBP > 100 mm Hg) and one case in the HDDB group (SBP > 160 mm Hg) were non-compliant to the study protocol because the patients’ blood pressure exceeded what was stated in the exclusion criteria. The protocol breach did not result in patient harm, and it was reported to the CIRB with the implementation of a preventive action plan. All three patients were included in the analysis.

**Figure 2:**
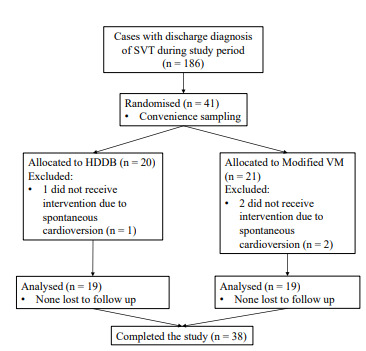
Patient flow diagram. *SVT*, supraventricular tachycardia; *HDDB*, head down deep breathing; *VM*, Valsalva manoeuvre.

In total, 38 patients were analyzed: 19 (50%) in the HDDB group and 19 in the modified VM group (Figure 2). Table 1 displays the baseline characteristics of the sample. Despite the small number of patients, the baseline features appeared sufficiently similar. All patients had initial rhythm SVT, and patients in both groups had comparable initial mean heart rate.

For the primary study outcome, cardioversion was achieved in six patients (31.6%) in the HDDB group and seven patients (36.8%) in the modified VM group. Four (21.1%) patients in the HDDB group and five (26.3%) patients in the modified VM group cardioverted during the first attempt. Modified VM was more likely to have successful cardioversion as compared to HDDB, but the association was not significant (OR: 1.26, 95% CI, 0.33, 4.84; *P* = 0.733]; a similar result was observed for successful cardioversion at first attempt (OR: 1.34, 95% CI, 0.30, 6.02; *P* = 0.703).

A total of 17 (89.5%) patients from the HDDB group and 14 (73.7%) from the modified VM group, respectively, did not encounter any adverse effects. However, patients who received the modified VM had three times the odds of experiencing an adverse effect as compared to HDDB, but the association was not significant (OR: 3.04, 95% CI, 0.51, 18.11; *P* = 0.223). There were no serious adverse events, such as cardiac arrest or malignant arrhythmia, which would have required immediate resuscitation among the patients in both groups. Minor adverse effects such as nausea, sweatiness, and giddiness were reported (Table 2).

Two patients in the modified VM group had chest pain. One of them had pain during the first attempt which cardioverted the SVT successfully. Post-conversion ECG did not reveal acute ST-segment changes and he was admitted for observation. The other patient had pain during the second attempt but was able to complete the study without successful cardioversion. The attending doctor then attempted standard VM which also failed, and eventually intravenous (IV) adenosine was successful. The patient was subsequently discharged without any adverse outcome.

Twenty-five (65.8%) patients remained in SVT at the end of the study. Table 3 describes the treatment methods used when the study interventions had failed. Six patients received crossover treatment. All of them underwent the treatment immediately when the study ended as part of usual care. One patient from the modified VM group who received HDDB was successfully cardioverted. The most common drug therapy used was IV adenosine. It demonstrated a high success rate with 17 out of 20 patients (85%) who received this treatment successfully cardioverted. Eventually, all except two (5.3%) patients were cardioverted at the ED. One patient was given IV amiodarone and oral bisoprolol and was admitted for further management. The other patient was discharged against medical advice. The majority of the patients, 73.7% (n = 28) were discharged. Ten patients were admitted; their mean age was 61.1 years.

## DISCUSSION

Vagal manoeuvres such as the modified VM slow down conduction in the atrioventricular (AV) node, resulting in the termination of AV nodal dependent reentrant tachycardias such as AVNRT and AVRT, which constitute the majority of regular narrow complex tachycardias. In the ED, the VM is commonly used on patients presenting with SVT. Even in the absence of a manometer, one can use a 10 milliliter Terumo syringe (Terumo Medical Canada Inc., Vaughan, Ontario, Canada) to provide the required 40 mm Hg pressure and achieve the standardised strain needed in a good VM.^6^ A Cochrane systematic review did not find sufficient evidence to support or refute the effectiveness of VM for termination of SVT.^7^ However, Appelboam et al found that postural modification to the standard VM (REVERT study) had a high success rate of 43% and recommended it as routine first treatment for SVT patients.^4^ Another vagal manoeuvre, the carotid sinus massage is less commonly performed due to the risk of cerebrovascular accident and, in rare instances, ventricular tachycardia.^8^ It should be avoided in patients with previous transient ischaemic attack or stroke, and in patients with carotid bruits.^2^ A study comparing the VM and carotid sinus massage for SVT treatment found similar success rates for the two methods.^9^

We describe the HDDB technique which does not require the patient to execute a VM, removing the need to rely on the patient’s effort and ability to deliver a good quality VM repeatedly. It also avoids the issue of the lack of standardisation as to how the VM is performed.^10^ Waxman et al have previously described the capacity of deep inspiration and dependent body position to terminate tachycardia in 11 patients with recurrent paroxysmal SVT.^11^ Drawing from their experience, we have reported success with the HDDB technique.^3^ It is believed that during inspiration, pulmonary stretch receptors inhibit the efferent vagal tone. By deep breathing in a head down position, venous return to the heart is increased and contributes to a gradual elevation of blood pressure. During expiration, the removal of pulmonary stretch enhances the efferent vagal tone which is also accentuated by the baroreceptors due to raised blood pressure. From our experience, HDDB patients are able to follow our instructions well, to draw full deep breaths and hold their breaths while we count with them at the bedside.

In our study, both HDDB and modified VM showed good success rates. The incidence of cardioversion with HDDB at 31.6% was higher than our anticipated value of 20%. Unfortunately, the minimum number of subjects that needed to be enrolled was not reached; so there was insufficient statistical power to analyse the data for a treatment effect. Both methods were found to be safe and did not result in any major adverse cardiovascular events. The most common choice of drug in accordance with national resuscitation guidelines was IV adenosine, which was effective at cardioverting most patients who failed vagal manoeuvres safely. We conclude that HDDB is a simple technique which is a useful addition to the current repertoire of vagal manoeuvres for the acute ED management of stable SVT. Further studies are needed to outline its safety and clinical efficacy.

## LIMITATIONS

Limitations include a small sample size which prevented effective comparison of treatment effects between the two techniques. Additionally, due to the convenience sampling method, not every patient who presented with paroxysmal SVT was assessed for eligibility. This could have led to selection bias. Finally, adverse effects were reported by patients and not consistently verified by the investigators and CRCs with a checklist. This may potentially have resulted in under-reporting.

## CONCLUSION

Our study found that both head down deep breathing technique (31.6% success) and modified Valsalva manoeuvre described by the REVERT study (36.8% success) were effective in cardioverting ED patients with supraventricular tachycardia. Both methods were safe and did not result in any major adverse cardiovascular events. This suggests that the HDDB method is a simple technique and a useful addition to the current repertoire of vagal manoeuvres for the acute management of stable SVTs, especially in low-resource settings. However, this is a preliminary study with small numbers, and further studies are needed to outline its safety and clinical efficacy.

## Figures and Tables

**Table 1:** Baseline characteristics and initial vital signs.

Characteristic	Head down deep breathing group (n = 19) n (%)	Modified Valsalva manoeuvre group (n = 19) n (%)	*P* value
Gender			
Male	8 (42.1)	11 (57.9)	0.330
Female	11 (57.9)	8 (42.1)	
Race			
Chinese	7 (36.8)	12 (63.2)	0.330
Malay	6 (31.6)	2 (10.5)	
Indian	1 (5.3)	1 (5.3)	
Others	5 (26.3)	4 (21.1)	
Age in years, mean (SD)	50.2 (19.0)	54.5 (14.3)	0.433
BMI, n	6	9	0.824
Mean (SD)	24.8 (2.4)	24.2 (5.9)	
History of			
Diabetes mellitus	5 (26.3)	2 (10.5)	0.405
Hypercholesterolaemia	5 (26.3)	4 (21.1)	1.000
Stroke, transient ischaemic attack	0 (0.0)	1 (5.3)	1.000
Atrial fibrillation	1 (5.3)	0 (0.0)	1.000
Initial vital signs			
SBP, mean (SD)	123 (18.9)	126 (17.4)	0.583
DBP, mean (SD)	84 (12.8)	83 (11.7)	0.875
Heart rate, mean (SD)	174 (23.6)	173 (23.5)	0.880
Initial ECG			
SVT	19 (100.0)	19 (100.0)	NA
Values reported as mean (+/− SD) or n (%).			

*BMI*, body mass index; *SD*, standard deviation; *SBP*, systolic blood pressure; *DBP*, diastolic blood pressure; *ECG*, electrocardiogram; *SVT*, supraventricular tachycardia.

**Table 2:** Primary and secondary outcomes.

	Head down deep breathing group (n = 19) n (%)	Modified Valsalva manoeuvre group (n = 19) n (%)	Odds ratio (95% CI) REF = HDDB	*P* value
Primary outcomes				
Successful cardioversion	6 (31.6)	7 (36.8)	1.26 (0.33, 4.84)	0.733
Successful cardioversion (at first attempt)	4 (21.1)	5 (26.3)	1.34 (0.30, 6.02)	0.703
Un-sustained cardioversion observed	3 (15.8)	1 (5.3)		
Secondary outcomes				
Adverse effects	2 (10.5)	5 (26.3)	3.04 (0.51, 18.11)	0.223
Types of chest pain/discomfort	0 (0.0)	2 (10.5)		
Nausea	0 (0.0)	2 (10.5)		
Increased palpitation	0 (0.0)	1 (5.3)		
Sweatiness	1 (5.3)	0 (0.0)		
Giddiness	1 (5.3)	0 (0.0)		
Serious adverse events (cardiac arrest, malignant arrhythmia)	0 (0.0)	0 (0.0)		
No adverse effect	17 (89.5)	14 (73.7)		

*CI*, confidence interval; *REF*, referecnce; *HDDB*, head down deep breathing.

**Table 3:** Treatment methods used and the success rates when study interventions failed.

*Treatment methods used	Head down deep breathing group (n = 13) n (%)	Modified Valsalva manoeuvre group (n = 12) n (%)	*P* value
†Crossover to modified VM or HDDB	5 (38.5)	1 (8.3)	0.160
Successful cardioversion	0 (0.0)	1 (100.0)	
IV adenosine	11 (84.6)	9 (75.0)	0.645
Successful cardioversion	10 (90.9)	7 (77.8)	
Carotid massage	3 (23.1)	2 (16.7)	1.000
Successful cardioversion	1 (33.3)	1 (50.0)	
Standard VM	2 (15.4)	2 (16.7)	1.000
Successful cardioversion	1 (50.0)	1 (50.0)	
IV verapamil	0 (0.0)	1 (8.3)	1.000
Successful cardioversion	0 (0.0)	1 (100.0)	

* Total number of treatment methods exceed the number of patients because several patients needed more than one method for cardioversion. Two patients did not have cardioversion to sinus rhythm.

† All patients who received crossover treatments had it immediately when the study ended as part of usual care.

*VM*, Valsalva manoeuvre; *HDDB*, head down deep breathing; *IV*, intravenous.
